# A Tablet-Based Interactive Movement Tool for Pediatric Rehabilitation: Development and Preliminary Usability Evaluation

**DOI:** 10.2196/10307

**Published:** 2018-11-26

**Authors:** Danielle Levac, Helene M Dumas, Waleed Meleis

**Affiliations:** 1 Department of Physical Therapy, Movement and Rehabilitation Sciences Northeastern University Boston, MA United States; 2 Medical-Rehabilitation Research Center Franciscan Hospital for Children Brighton, MA United States; 3 Department of Electrical and Computer Engineering Northeastern University Boston, MA United States

**Keywords:** equipment design, rehabilitation, pediatrics, tablets, software

## Abstract

**Background:**

Motivating interactive tools may increase adherence to repetitive practice for children with disabilities, but many virtual reality and active video gaming systems are too challenging for children with significant needs.

**Objective:**

The objective of this study was to develop and conduct a usability evaluation of the Fun, Interactive Therapy Board (FITBoard), a movement toy bridging digital and physical interactions for children with disabilities.

**Methods:**

The FITBoard is a tablet app involving games controlled by hand, head, or foot touch of configurable, wired surfaces. Usability evaluation involved a cognitive walkthrough and think-aloud processes. Participants verbalized aloud while completing a series of 26 task actions involved in selecting a game and configuring the FITBoard to achieve the therapeutic goal. Therapists then responded to questions about usability perceptions. Unsuccessful actions were categorized as goal or action failures. Qualitative content analysis supported understanding of usability problems.

**Results:**

Participants included 5 pediatric physical therapists and 2 occupational therapists from 2 clinical sites. Goal failure was experienced by all participants in 2 tasks, and action failure was experienced by all participants in 2 tasks. For 14 additional tasks, 1 or more patients experienced goal or action failure, with an overall failure rate of 69% (18 of 26 tasks). Content analysis revealed 4 main categories: hardware usability, software usability, facilitators of therapy goals, and improvement suggestions.

**Conclusions:**

FITBoard hardware and software changes are needed to address goal and action failures to rectify identified usability issues. Results highlight potential FITBoard applications to address therapeutic goals and outline important practical considerations for product use by therapists. Subsequent research will evaluate therapist, parent, and child perspectives on FITBoard clinical utility when integrated within regular therapy interventions.

## Introduction

Children and adolescents with physical or developmental disabilities participate in rehabilitation to learn new motor skills, improve existing skills, and support capacity for self-care and independent living [1-3]. Motor learning requires abundant, challenging, progressive, varied, and feedback-rich practice opportunities to elicit meaningful change [4]. Providing these intervention characteristics is a major consideration in rehabilitation planning [5-7]. Therapists must select activities that are customizable to individual abilities and goals and that sustain children’s motivation to engage in challenging and repeated practice.

Enhancing and sustaining children’s motivation is important for rehabilitation because motivation is an affective state that may mediate the functional brain changes (ie, neuroplasticity) that influence motor learning [8]. Motivation is a child characteristic thought to influence changes in motor ability for children with cerebral palsy [9], although no empirical link has been made between motivation and rehabilitation effectiveness in pediatric populations [10]. Therapists can enhance motivation by involving the child in selection of therapeutic tasks that are relevant to his or her interests and goals [11].

Interactive digital screens, including hand-held tablets, active video games (AVGs), and fully immersive 3D virtual reality (VR) systems, have recently become accessible, motivating therapeutic task options for children [12]. VR and AVGs encourage children to interact with onscreen simulations using body movements. The therapeutic advantages include repetitive practice, customized difficulty levels, metrics to track progress, and the potential for telerehabilitation [13-15]. Inexpensive, off-the-shelf AVGs, such as the Nintendo Wii or the Microsoft Kinect, however, may be too challenging for young children, children with perceptual or cognitive impairments, or children with more severe physical or cognitive limitations [16]. VR systems designed specifically for rehabilitation use can address some of these barriers but may have greater cost and training requirements.

In contrast to full body movement interaction, tablets are popular therapy tools used to stimulate fine motor movements and cognitive processes through a variety of games and apps [17]. These touch devices are portable, accessible, and fairly inexpensive. Children with disabilities, including preschoolers, can quickly become competent with these devices [18]. The body of evidence on whether the use of touch screens can support cognitive learning for children with disabilities is small, primarily focusing on children with autism spectrum disorder [18,19]. For children with fine or gross motor impairments, alternative interface modalities such as switches and push buttons are recommended to replace the *swipe and touch* movements requiring control and force regulation to interact with the screen [20].

In an attempt to build on the benefits and address the challenges of AVGs and tablet use in children with disabilities, we developed an alternative interface modality called the Fun, Interactive Therapy Board (FITBoard), a movement toy bridging digital and physical interactions. The FITBoard is a tablet app involving custom-designed games in which tablet screen touch is replaced by hand, head, or foot touch of configurable, wired surfaces. The FITBoard was designed to help children practice movement skills during physical or occupational therapy. Children reach and touch keys on the FITBoard panels to control the games on the tablet screen. The games are designed with the intent to meet the needs of children and youth at a variety of cognitive and physical abilities and provide challenging, progressive, varied, and feedback-rich practice opportunities to address therapeutic goals and elicit functional change.

Undertaking usability testing is important because many new interactive health care apps remain unused when they do not meet the needs of users [21]. Usability evaluation is part of a user-centered design process to understand effectiveness, efficiency, and appeal of a tool for users [22]. Usability testing provides the opportunity for individuals who will ultimately be users of the product to participate in its refinement [23]. The objective of this study was to describe development and preliminary usability evaluation of the FITBoard among physical and occupational therapists at 2 pediatric clinical sites.

## Methods

### Research Design

This usability study was approved by the institutional review board at the 2 clinical sites, Franciscan Children’s Hospital and Spaulding Rehabilitation Hospital, Boston, MA, USA.

### Participants

Pediatric physical therapists (PTs) and occupational therapists (OTs) were recruited through volunteer sampling to participate in the usability evaluation. Therapists were invited to attend information sessions and were provided with a description of the project objectives, procedures, benefits, and risks. Therapists who were interested in taking part in the study provided written informed consent before participation. At 1 site, 5 of the 6 eligible therapists agreed to participate. At the second site, 2 of the 12 eligible therapists agreed to participate.

### Fun, Interactive Therapy Board Development

Initial development of the FITBoard was informed by gathering perspectives on desired device characteristics from PTs and OTs through an informal needs assessment at the Spaulding Rehabilitation Institute before study initiation. Through informal discussion with the principal investigator (PI; DL), 5 therapists (3 PTs and 2 OTs) expressed the need for a device with the following characteristics: low cost; gaming-based; flexible to address varied physical and cognitive impairments; usable through hand, foot, or head movements; durable for energetic physical play; involving touch of different surfaces; constructed from easily sanitized materials; and capable of tracking patient progress.

A team of electrical, computer, and mechanical engineering undergraduate and graduate students at Northeastern University (Boston, MA, USA) was led by the project PIs to produce a prototype FITBoard. The individual PI’s experience included expertise with AVGs, considerable experience leading student groups in low-cost device construction for individuals with disabilities, or clinical expertise in pediatric rehabilitation. Over a 12-month period, various iterations were constructed and programmed to match the requested characteristics.

**Figure 1:**
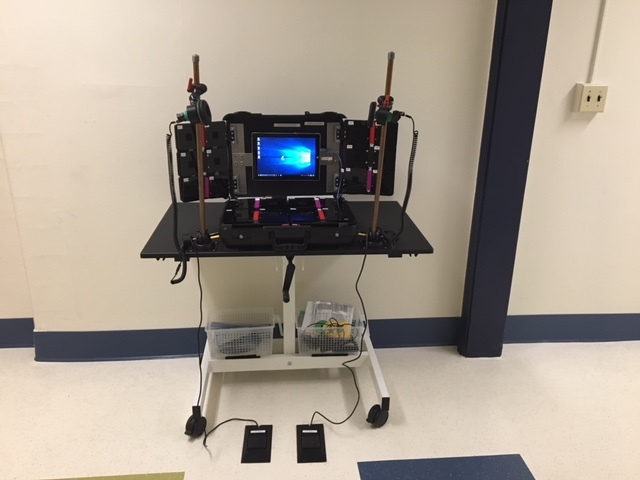
The Fun, Interactive Therapy Board (FITBoard).

The resultant FITBoard (see Figure 1) is a physical interface running a tablet app that displays games controlled by hand, head, or foot touch. It operates via panels that have 3” × 5” keys with pressure switches and resistors that provide differing analog inputs to an Arduino microcontroller. The key covers hinge from 1 side allowing the pressure switch to be activated regardless of where on the panel covering it is pressed. Each panel also has a Velcro component to enable different materials representing cues for game actions or other sensory-stimulating touch surfaces to be attached.

The interface is a box like design with folding panels that extend from a case resembling a hard-shell luggage product. Top folding panels are made of acrylic and friction hinges. Bottom panels slide in and out using guide rails made from aluminum extrusions. The top panels are double-sided and fold out to keep the lid light, whereas the bottom panels slide for extra stability. Additional panels are arranged below the sliding path so that the device can be used with the bottom panels extended or kept inside the case. There are removable head and foot controls that can be positioned to accommodate user needs. The FITBoard rests on a wheelchair-accessible height-adjustable wheeled desk to accommodate users of different heights.

The app is displayed on a Microsoft surface tablet, chosen because it has a universal serial bus (USB) port for the Arduino to communicate button press signals into the game. The 7 custom-built games are built in Unity3D and scripted in C#. The games are appropriate for a variety of ages and children with varying cognitive abilities. For example, in the *Paint a Picture* game, key presses result in a splash of color on the screen. The user can try to cover the screen with paint splashes of varying colors within the preset time limit. In the *Drive the Car* game, users press keys corresponding to direction and speed to steer a car through a course of varying obstacles and difficulties. Each game incorporates visual and auditory effects; offers multiple challenge levels; can be played with head, foot, or hand controls; and provides feedback to the user about game play success.

**Figure 2:**
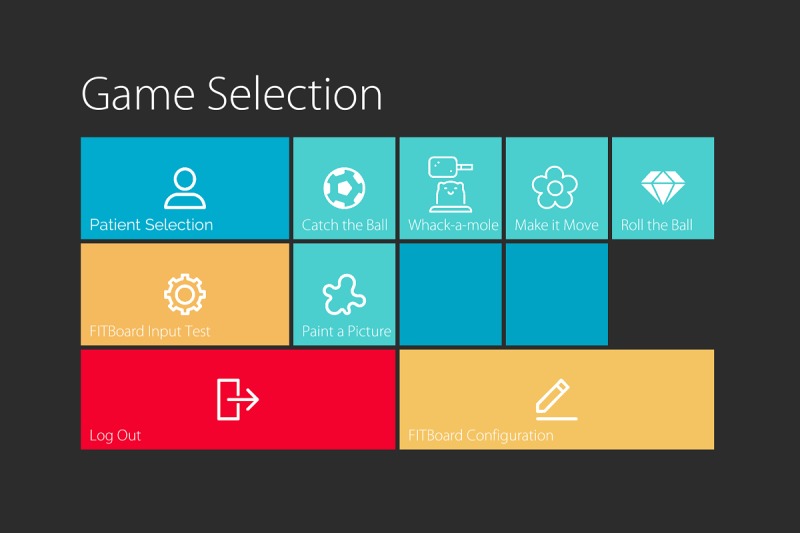
The Fun, Interactive Therapy Board (FITBoard) game selection screen.

To use the FITBoard, the therapist configures the physical device to the target therapy goal or goals (eg, positioning the panels, so the child has to reach across his or her body; using foot controls to facilitate stepping). The therapist then signs in to the app, selects an existing client or adds a new client, and selects a game to play (see Figure 2). Next, the therapist selects the specific FITBoard keys that he or she would like the child to use to play the game and adds a laminated paper (eg, arrows and colored circles) or other material as a cue to indicate that key’s action. Once at a game menu, settings such as game difficulty (eg, speed) and time can be selected or the user can choose to simply continue with previously used settings. Game play data are saved on the tablet.

### Usability Evaluation Methods

Usability evaluation was undertaken with cognitive walkthrough (CW) [22-24] and think-aloud (TA) [25] approaches. CW is a form of task analysis that enables evaluation of early prototypes to uncover possible errors in design that would interfere with the user’s ability to learn how to use the system and conduct the required tasks [22]. CW involves moderator observing users completing a walkthrough of tasks required to use the system predivided into single actions. In this study, users were required to *set up a new therapist and client account*; *select, set up, and play a game*; *exit the application*; *identify a client goal and provide a rationale for FITBoard use*; *configure the FITBoard for the identified client*; and *select and implement a game for that client*. Table 1 depicts the steps for each task. As the moderator observes the participant moving through the tasks, he or she records observations as to whether the user is successful or whether there are *goal failures* (the user accomplishes the wrong thing) or *action failures* (the user would like to perform the correct action but does not know how) [24].

The TA method is a widely used usability evaluation method often employed in conjunction with CW [25,26]. It involves asking potential users to *think aloud* as they interact with the product. TA is complementary to CW because it focuses on cognitive processes relevant to task completion. It is considered the gold standard because it supports greater understanding of the problems that users are having with interaction [25]. Sessions are audio-recorded, and the participant is encouraged to speak constantly as if alone in the room. He or she is given nonobtrusive reminders if they fall silent; otherwise, the moderator does not interfere.

### Study Procedures

CW and TA procedures occurred during 1-hour individual audiotaped sessions led by 1 of 2 moderators (study investigators DL or HMD) in private testing rooms. Participants began by following a series of printed actions to set up a new therapist and client account, select and play a game, and close down the FITBoard app. They were then asked to describe a hypothetical or real client and a therapeutic goal for FITBoard use and subsequently complete a series of task actions involved in selecting a game and configuring the FITBoard to achieve the therapeutic goal. Moderators observed, documented, and categorized actions during the CW as goal failures (user tries to accomplish the wrong thing) or action failures (user would like to perform the correct action but does not know how). After completing the CW and TA, participants responded to 4 structured questions about FITBoard use. The 4 questions were specific to features therapists appreciated or found frustrating about FITBoard use as well as eliciting suggestions for FITBoard improvement and any other comments the therapist wished to share.

**Table 1:** Cognitive walkthrough results.

Task and task description		Goal failures, n (%)	Action failures, n (%)	Successes, n (%)
**Set up a new therapist and client account**				
	Turn on tablet	0 (0)	5 (71)	2 (29)
	Attach keyboard and type in password	0 (0)	4 (57)	3 (33)
	Plug in USB^a^	0 (0)	2 (29)	5 (71)
	Locate FITBoard^b^ icon	0 (0)	2 (29)	5 (71)
	Sign up for new therapist account	3 (33)	0 (0)	4 (57)
	Add a new client	0 (0)	0 (0)	7 (100)
**Game selection, set up, and play**				
	Use game descriptions to select a game	7 (100)	0 (0)	0 (0)
	Use the interface to select 4 keys	0 (0)	7 (100)	0 (0)
	Apply contact material to keys	0 (0)	4 (57)	3 (33)
	Play game	0 (0)	2 (29)	5 (71)
**Exit the app**				
	Log-out of app	0 (0)	3 (33)	4 (57)
	Remove contact material	0 (0)	0 (0)	7 (100)
	Turn off tablet	0 (0)	3 (33)	4 (57)
**Identify a client^c^**				
	Identify a client	0 (0)	0 (0)	5 (100)
	Identify a task or activity	0 (0)	0 (0)	5 (100)
	Provide rationale for how FITBoard will assist in that task or activity	0 (0)	0 (0)	5 (100)
**Configure FITBoard for client^c^**				
	Log-in to FITBoard app using existing therapist and patient ID	0 (0)	1 (20)	4 (80)
	Identify patient starting position	0 (0)	0 (0)	5 (100)
	Open, close, or slide top or bottom panels	0 (0)	5 (100)	0 (0)
	Add head or foot controls	0 (0)	4 (80)	1 (20)
	Raise or lower the desk	0 (0)	0 (0)	5 (100)
**Select and implement game for client^c^**				
	Use game descriptions to select a suitable game	5 (100)	0 (0)	0 (0)
	Use the interface to select 4 keys	0 (0)	0 (0)	5 (100)
	Apply contact material to keys	0 (0)	2 (40)	3 (60)
	Select appropriate game settings	2 (40)	3 (60)	0 (0)
	Progress, modify, or change the activity	0 (0)	0 (0)	5 (110)

^a^USB: universal serial bus.

^b^FITBoard: Fun, Interactive Therapy Board.

^c^Of 7 participants, 5 completed these tasks.

### Analyses

Goal and action failures from the CW were summarized with descriptive statistics. TA process and interview question audio-recordings were transcribed. One PI (DL) undertook summative qualitative content analysis [27] focusing on words and content used by participants. Content was interpreted to specifically identify usability problems and to summarize suggestions for improvement.

## Results

### Participants

In this study, 5 pediatric PTs and 2 OTs (mean 19.3 years of clinical experience, range 3-33 years) participated from 2 in-patient pediatric rehabilitation clinical sites. Overall, 3 therapists (2 OTs and 1 PT) had participated in the previously described informal needs assessment.

### Cognitive Walkthrough

Goal failure (user accomplishes the wrong thing) was experienced by all participants in 2 tasks (*using game descriptions to select a game* and *select appropriate game settings*), whereas action failure (user would like to perform the correct action but does not know how) was experienced by all participants in 3 tasks (*select game keys*, *open or close or slide FITBoard panels*, and *select appropriate game settings*). In total, 14 additional tasks experienced action failures by 1 or more participants. There was an overall rate of 31% tasks completed successfully by at least one participant (8/26 tasks), and 69% failed tasks (either goal or action failure) by at least two participants (18/26 tasks). Table 1 provides results of the CW process. Table 2 provides examples of goal and action failures experienced.

In total, 5 of the 7 therapist participants identified a hypothetical or real client to consider during the CW. Identified client impairments included reduced strength and altered muscle tone in 1 upper extremity (n=1), hemispatial neglect (n=1), or static and dynamic standing balance difficulties (n=3). Therapists provided rationales for FITBoard use, including increasing awareness and movement of the affected upper extremity, engaging the child while maintaining desired periods of static standing balance, and encouraging stepping outside of the base of support to improve dynamic standing balance. Therapists reported that they would position their hypothetical or real client in sitting (n=1), standing when using the 2 foot controls (n=2), and standing without the foot controls (n=2).

**Table 2:** Examples of goal (user accomplishes the wrong thing) and action (user would like to perform correct action but does not know how) failures.

Task	Goal or action failures
Using game descriptions to select a game	Goal: Participants did not *long press* on the game icon to bring up the game descriptions
Use the app interface to select game keys	Action: Participants were not able to understand how icons represented actions in the game
Open or close or slide FITBoard^a^ panels	Action: Participants were not sure how much force to use to move or slide the panels and in what direction
Select appropriate game settings	Goal: Participants missed the *game settings* option on the screen and did not select it
Attach keyboard and type in password	Action: Participants did not recognize magnetic interface to attach keyboard
Plug in USB^b^	Action: Participants did not know where to plug in the USB
Locate FITBoard icon	Action: FITBoard icon was small and participants had difficulty locating it on the screen
Sign up for new therapist account	Goal: Participants tried to *log-in* without first signing up for a new account
Apply contact material to keys	Action: Participants were not clear where to find contact materials, which ones they should use
Play game	Action: Participants did not see results of key presses on screen because not pressing correct keys at correct time for the game interface
Log-out of app	Action: Participants quit without logging out
Turn off tablet	Action: Participants were not sure how to turn off the tablet
Log-in to FITBoard app using existing therapist and patient ID	Action: Participant tried to sign up instead of log-in
Add head or foot controls	Action: Participants were not sure where to plug in head or foot controls

^a^FITBoard: Fun, Interactive Therapy Board.

^b^USB: universal serial bus.

### Think Aloud and Interviews

Content analysis of the TA and interview transcripts revealed 4 categories: hardware usability (ie, FITBoard fragility), software usability (ie, key configuration and game settings), facilitators of therapy goals, and suggestions for improvement.

#### Hardware Usability

All therapists expressed concern about the physical appearance of the FITBoard, reporting they were apprehensive about its fragility and durability. Therapists reported being uncertain whether the force required to move and slide the FITBoard panels would cause the panels to break and were concerned that pediatric clients would pull the exposed wires. For example, one therapist said:

...you know how when you’re working with technology and you have to guide people how hard they can hit without breaking the machine? So, I’m thinking at what point do I tell a kid “don’t hit that so hard”?...also how easily they [the panels] pop off...so my concern with this is with kids, no matter how much you tell them “I got you,” if they go to fall, they will grab onto this, and I just feel like this is something I wouldn’t want to pull on, so it almost like narrows who my population who I think would benefit from it.

Another therapist said:

I know this is a prototype but I would hope that the permanent thing is a little sturdier...too many wires that a patient could inadvertently pull off, break off, knock over...someone with strong tone or any sort of spasticity...it felt very fragile.

A third therapist commented:

I’m not sure about the durability and feasibility of it, meaning, putting it together, setting it up...I’d be afraid that it wasn’t going to hold up very well...sliding [the panels] in, sliding [the panels] out.

Additional hardware concerns expressed by the therapists related to the sensitivity of the panel keys:

It’s nice that you have that sensitivity [to touch the keys] but on the other hand we have a lot of kids that don’t have that controlled movement...

Finally, the inability to mount the tablet above the FITBoard to encourage children to raise their head to look at the screen was reported as problematic.

#### Software Usability

Software usability included challenges interacting with the FITBoard app interface on the tablet. Participants found aspects of the app interface confusing, particularly the game selection screen where they were not able to complete the task of viewing the game description. When shown this task action following the CW, participants expressed concern that without a picture of the game, it was difficult to understand the description. For example, with respect to the *Whack a Mole* game, 1 participant said:

Well I don’t know what the holes [where the Moles come up] look like...are they in a grid? Are they on top of each other, because of the up [and] down [buttons]? Without seeing the screen of what the holes look like, I don’t know what that means. I’m not sure for top and bottom [keys], what I’d choose.

In the key configuration screen where participants select specific keys on the FITBoard to interface with the game, participants disliked the incongruity between the visual representation of the key icons on the FITBoard panels and their actions in the game reporting it was very confusing to understand which key undertook which game action. Participants also reported challenges locating and then understanding how to set the game difficulty levels, including game duration and speed.

One participant was concerned that the games may not be visually appealing for her clients given the high-quality graphics of the media with which children typically interact:

...I would love to see what the kids think [about these games] because now video games have so many components to them and they are so animated and dynamic, and they have music and they have sounds and they can be more complex. So depending on the age of the kids and their cognitive abilities, I don’t know how they would like this [device]... it would really vary. Kids used to other games might not be thrilled with this [device] and then the older kids and teens might not like the games.

#### Facilitators of Therapy Goals

Participants appreciated the many options (eg, head switches, foot pedals, and panel positions) to elicit movement and the selection of tactile touch contact materials for interaction (eg, plastic arrows and toy animals) as well as the many opportunities to individualize the intervention. For example, one therapist said:

I think it would be good for kids that like video games...maybe they are working on gross movement...a child with hemiplegia – it would be a fun game to get reach to the side, and yet it’s not a lot of fine motor so you can get them to do some gross moves with their upper extremity.

Another therapist described potential use of the FITBoard with a particular child, stating:

I can make him reach out of his base of support, I could make him tap his foot and I think it would engage him as well because he loves video games.

Another therapist focused on the ability to interact with the game using only simple head movements, saying it would be appropriate for a current patient because:

...she has very poor head control so something we were trying to do today in rehab we were drawing tic-tac-toes and trying to get her to look up and get her head up. So this could be something for her that I use this for.

This was echoed by a second therapist, who said:

I like that you can...make it so specific to the patient you have...You do have something for even if it’s for something like head control...because we do get a significant amount of people that, that is a serious thing we are working on and it’s hard to make that fun sometimes.

#### Suggestions for Improvement

There were multiple specific suggestions for improving the FITBoard hardware and software interfaces. Hardware suggestions included increasing the stability of the head and foot controls, covering the microcontroller to protect it from exposure to cleaning fluids, and increasing the mobility of the top and side panels so they could be positioned higher and surround the user.

Software improvement suggestions included adding a pause feature to the games:

...I get so many interruptions at random times in the session...work with kids that need that closure...they want to make sure you can pause and finish that game and get that score, or like they won’t be able to listen to what a nurse has to say or take a medication, unless they can pause that game.

One therapist suggested a more intuitive way to access and store the touch materials, saying:

...they would benefit from being labeled so[it would be] easier to put your hands on stuff...

In addition*,* further touch materials were suggested, including materials to facilitate use by clients with limited fine motor control:

I think that for her [the patient] I’d work on some grasp and I don’t think she could get a good grasp and fall off of it. So, something she could rest her hand here and squeeze a little I mean depending on their hand skills.

Additional suggestions included having the games to provide more feedback about success or error rate and including games that required only 1 or 2 keys rather than 4 keys to play. Suggestions were also made to improve the user instructions with additional details, add a game description sheet to accompany the FITBoard, and round off sharp edges of the laminated paper pieces that attach to the keys.

## Discussion

### Principal Findings

The objective of this study was to develop and conduct a usability evaluation of the FITBoard, a movement toy bridging digital and physical interactions for children with disabilities. Usability was evaluated through CW and TA methods to enable identification of problematic tasks involved in using the FITBoard and identify areas for improvement.

The 69% overall goal and action failure rate in this study was similar to others in the literature. Peute et al [26] undertook a CW and TA evaluation of a new Web-based laboratory test ordering tool with 7 participants, finding that 16 of 25 (16/25, 65%) actions resulted in goal or action failures. Valdes et al [28] used CW and TA to evaluate 2 newly developed motion-tracking rehabilitation therapeutic tools. They reported that 69.5% of the actions evaluated in their sample of 11 therapists had some element of failure but did not classify failures into goal or action components [28].

Our testing situation was unique because it focused on evaluating usability of both novel hardware and software interfaces, which differs, for example, from usability testing of a new website where users could be expected to be familiar with general layout and functioning of a keyboard, mouse, and monitor. The CW and TA processes illuminated usability problems and flaws in the process of using the FITBoard from the beginning to the end that led to errors for some or all participants. The primary usability problems included structural issues with the FITBoard that prevented users from being comfortable interacting with the device (ie, opening and closing panels and attaching foot and head controls). Other problems were related to lack of clarity in FITBoard software interactions (eg, how to select keys to play the game and how to find game descriptions before selecting a game). The results from CW and TA identified problematic tasks that must be addressed before therapists are able to test the FITBoard with children and families.

Despite these limitations, participants easily identified a client and functional goal that would be relevant to FITBoard use. In addition, they appreciated the diversity of options that the FITBoard provides to motivate and engage children in maintaining upright head control, which was identified as a priority in the informal needs assessment. Therapists appeared to view the FITBoard as relevant to the goals for the patients on their caseloads and provided valuable information to direct changes to the FITBoard before evaluation of its clinical utility.

Rehabilitation therapists have access to technological options, including VR, active video gaming, and other tablet apps that are commercially available and/or developed specifically for rehabilitation. As such, it is difficult for housemade systems and games to compete with commercially available choices in terms of aesthetic appeal or intuitive user interfaces. We know from barriers and facilitators assessments in the field of AVG use that the main barriers to introduction for these new technologies are practical difficulties such as cost, adequate space for use, and time to learn how to use, including how to choose specific games or apps most relevant to patients’ goals [16,28,29]. Therapists wanted a tool that would work for children with more significant physical or cognitive impairments and for a younger age range than what is typical for AVG use.

The FITBoard has the potential for use with young children and children with significant needs. However, the current model is larger than initially desired, given our initial goal of a device that could fold down to be stored in a briefcase-like fashion. We emphasized durability of individual materials used in the design but the overall device is more fragile than originally anticipated.

### Limitations

Although our development process began by soliciting input from therapists, it could have been more user-centered and iterative if it had taken place in close proximity to the therapists, allowing them to provide more regular input throughout the process. This did not occur because of the cost involved in transporting the FITBoard from the laboratory in which it was built to each clinical site as well as a reluctance to place an additional burden on therapists’ time. This limitation was evident, for example, in findings related to therapists’ recommendations about having panels extend higher and laterally to surround the participant, which might have been able to be implemented in early stages of construction.

The CW and TA processes were undertaken by authors DL and HMD, researchers known by therapists to be invested in FITBoard development. Despite assurances that all feedback was welcome, therapists may have felt uncomfortable expressing negative opinions about the device in their presence. In addition, the CW process has been criticized as being too rigid and, therefore, limiting the types of problems discovered [30]. The authors did not undertake traditional forms of qualitative data credibility analysis such as member checking or triangulation. Finally, the study is limited by a small sample, which may not have been sufficient to discover all usability problems and did not allow for comparisons between physical and occupational therapists in terms of their perspectives on the device. Although Bastien [23] suggests that 8 participants are sufficient for a TA process, there is no consensus on the number of participants required. In total, 2 of the 7 participants did not have time to complete the full CW. Moreover, there were only 2 OT participants. This is important because OTs may have different therapeutic rationale and interests in using this device. Including additional OTs might have led to the discovery of different usability problems [30].

### Next Steps

Study results are guiding changes to the FITBoard to address hardware and software usability issues. Our next steps are to reintroduce the revised FITBoard to the clinical sites and undertake a clinical utility study with therapists, children, and families to determine how FITBoard use addresses relevant therapeutic goals. Therapists will use the FITBoard on several occasions, recording their functional goals and perceptions of how FITBoard use was able to address the goal; therapists, parents, and children, as able, will complete standardized measures evaluating satisfaction, engagement, and motivation. Finally, we will conduct interviews with children, parents, and therapists to further identify barriers to and facilitators for FITBoard use. On the basis of the results, we can approach industry partners with respect to making changes to the FITBoard interface and app to support creation of additional, improved devices with a larger budget for construction and game development. We would then undertake longer-term feasibility and effectiveness research in home or school settings to understand the potential role of the FITBoard in therapeutic programs.

### Conclusions

The FITBoard is a newly developed, low-cost rehabilitation tool for movement skill practice that integrates the motivating attributes of video games with the functional, touch-based sensory input of traditional rehabilitation interventions. Usability testing methods (CW and TA) with a small sample of physical and occupational therapists revealed FITBoard hardware and software concerns, potential apps for therapy goals, and suggestions for improvement. FITBoard hardware and software changes are needed to address goal and action failures and respond to identified usability issues. Following these improvements, our goal is to produce an accessible, user-friendly, and low-cost product that can be integrated into school, home, or community programs to enhance practice dosage of functionally relevant movement skills for children and youth with disabilities.

## Abbreviations

AVG: active video game
CW: cognitive walkthrough
FITBoard: Fun, Interactive Therapy Board
OT: occupational therapist
PI: principal investigator
PT: physical therapist
TA: think-aloud
USB: universal serial bus
VR: virtual reality

## References

[1] Valvano J (2004). Activity-focused motor interventions for children with neurological conditions. Phys Occup Ther Pediatr.

[2] Majnemer A, Shikako-Thomas K, Lach L, Shevell M, Law M, Schmitz N, Poulin C, QUALA Group (2014). Rehabilitation service utilization in children and youth with cerebral palsy. Child Care Health Dev.

[3] Anaby D, Korner-Bitensky N, Steven E, Tremblay S, Snider L, Avery L, Law M (2017). Current rehabilitation practices for children with cerebral palsy: focus and gaps. Phys Occup Ther Pediatr.

[4] Gordon AM, Magill RA, Campbell SK, Palisano RJ (2012). Motor learning: application of principles to pediatric rehabilitation. Physical Therapy for Children. 4th edition.

[5] Kleim JA, Jones TA (2008). Principles of experience-dependent neural plasticity: implications for rehabilitation after brain damage. J Speech Lang Hear Res.

[6] Reid LB, Rose SE, Boyd RN (2015). Rehabilitation and neuroplasticity in children with unilateral cerebral palsy. Nat Rev Neurol.

[7] Palisano RJ, Begnoche DM, Chiarello LA, Bartlett DJ, McCoy SW, Chang H (2012). Amount and focus of physical therapy and occupational therapy for young children with cerebral palsy. Phys Occup Ther Pediatr.

[8] Wulf G, Lewthwaite R (2016). Optimizing performance through intrinsic motivation and attention for learning: The OPTIMAL theory of motor learning. Psychon Bull Rev.

[9] Bartlett DJ, Palisano RJ (2002). Physical therapists' perceptions of factors influencing the acquisition of motor abilities of children with cerebral palsy: implications for clinical reasoning. Phys Ther.

[10] Tatla SK, Sauve K, Virji-Babul N, Holsti L, Butler C, Van Der Loos HF (2013). Evidence for outcomes of motivational rehabilitation interventions for children and adolescents with cerebral palsy: an American Academy for Cerebral Palsy and Developmental Medicine systematic review. Dev Med Child Neurol.

[11] Majnemer A (2011). Importance of motivation to children's participation: a motivation to change. Phys Occup Ther Pediatr.

[12] Weiss PL, Tirosh E, Fehlings D (2014). Role of virtual reality for cerebral palsy management. J Child Neurol.

[13] Howcroft J, Klejman S, Fehlings D, Wright V, Zabjek K, Andrysek J, Biddiss E (2012). Active video game play in children with cerebral palsy: potential for physical activity promotion and rehabilitation therapies. Arch Phys Med Rehabil.

[14] Levin MF (2011). Can virtual reality offer enriched environments for rehabilitation?. Expert Rev Neurother.

[15] Ravi DK, Kumar N, Singhi P (2017). Effectiveness of virtual reality rehabilitation for children and adolescents with cerebral palsy: an updated evidence-based systematic review. Physiotherapy.

[16] Levac DE, Miller PA (2013). Integrating virtual reality video games into practice: clinicians' experiences. Physiother Theory Pract.

[17] Tatla SK, Shirzad N, Lohse KR, Virji-Babul N, Hoens AM, Holsti L, Li LC, Miller KJ, Lam MY, Van der Loos HF (2015). Therapists' perceptions of social media and video game technologies in upper limb rehabilitation. JMIR Serious Games.

[18] Fager SK, Burnfield JM (2014). Patients' experiences with technology during inpatient rehabilitation: opportunities to support independence and therapeutic engagement. Disabil Rehabil Assist Technol.

[19] Chmiliar L (2017). Improving learning outcomes: the iPad and preschool children with disabilities. Front Psychol.

[20] Esposito M, Sloan J, Tancredi A, Gerardi G, Postiglione P, Fotia F, Napoli E, Mazzone L, Valeri G, Vicari S (2017). Using Tablet Applications for Children With Autism to Increase Their Cognitive and Social Skills. J Spec Educ Technol.

[21] Howard AM, Park HW (2014). Using tablet devices to engage children with disabilities in robotic educational activities. J Technol Pers Disabil.

[22] Jaspers MW (2009). A comparison of usability methods for testing interactive health technologies: methodological aspects and empirical evidence. Int J Med Inform.

[23] Bastien JM (2010). Usability testing: a review of some methodological and technical aspects of the method. Int J Med Inform.

[24] Kushniruk AW, Patel VL (2004). Cognitive and usability engineering methods for the evaluation of clinical information systems. J Biomed Inform.

[25] Boren T, Ramey J (2000). Thinking aloud: reconciling theory and practice. IEEE Trans Prof Commun.

[26] Peute LW, Jaspers MM (2005). Usability evaluation of a laboratory order entry system: cognitive walkthrough and think aloud combined. Stud Health Technol Inform.

[27] Hsieh H, Shannon SE (2005). Three approaches to qualitative content analysis. Qual Health Res.

[28] Valdés BA, Hilderman CG, Hung CT, Shirzad N, Van der Loos HF (2014). Usability testing of gaming and social media applications for stroke and cerebral palsy upper limb rehabilitation. Conf Proc IEEE Eng Med Biol Soc.

[29] Glegg SM, Holsti L, Velikonja D, Ansley B, Brum C, Sartor D (2013). Factors influencing therapists' adoption of virtual reality for brain injury rehabilitation. Cyberpsychol Behav Soc Netw.

[30] Khajouei R, Zahiri Esfahani M, Jahani Y (2017). Comparison of heuristic and cognitive walkthrough usability evaluation methods for evaluating health information systems. J Am Med Inform Assoc.

